# The outcome of nonoperative treatment for adult humeral shaft fractures using a U-shaped slab in resource-limited settings: a prospective cohort study

**DOI:** 10.1186/s13018-024-04794-w

**Published:** 2024-05-28

**Authors:** Alexis Nshimiyimana, Jean de la Croix Allen Ingabire, Jean Claude Byiringiro, Basile Habumugisha, Jean Luc Mwizerwa

**Affiliations:** 1 College of Medicine and Health Science, University of Rwanda, Kigali, Rwanda; 2 Orthopedic Department, University Teaching Hospital of Kigali, Kigali, Rwanda; 3 Orthopedic Department, Rwanda Military Hospital, Kigali, Rwanda

**Keywords:** Humeral shaft fractures, Nonoperative management, U-shaped slab, Rehabilitation, Functional outcome

## Abstract

**Background:**

Humeral shaft fractures, constituting 3–5% of musculoskeletal injuries, are commonly managed conservatively using functional braces. However, this approach may not be feasible in resource-limited settings. This study aimed to evaluate the functional outcomes of nonoperative treatment for humeral shaft fractures in adults utilizing a U-shaped slab.

**Methods:**

This prospective study was conducted from August 2021 to August 2022 involving 16-year-old and older individuals who received nonsurgical treatment for humeral shaft fractures at public tertiary hospitals in Rwanda. The assessment focused on various functional outcomes, including alignment, union rate, range of motion, return to activities of daily living, and DASH score.

**Results:**

The study included 73 participants, predominantly males (73.9%), with a median age of 33 years. The union rate was high at 89.04%, and 10.96% experienced delayed union. Radial nerve palsy occurred in 4.11% of patients, but all the patients fully recovered within three months. Despite angular deformities during healing in the majority of participants, these deformities did not significantly impact functional outcomes. According to the international classification of disabilities, 77% of participants achieved a good functional grade.

**Conclusion:**

The conservative U-shaped slab method was effective at managing humeral shaft fractures. However, optimal results necessitate careful participant selection and comprehensive rehabilitation education. Implementing these measures can improve the overall success of nonoperative management.

## Introduction

### Background

Humeral shaft fractures significantly contribute to musculoskeletal injuries and are more common in men than in women; they constitute 3–5% of all adult fractures and impact 20% of humeral fractures in the adult population [1]. The incidence of humeral shaft fractures has a dual-peaked age distribution [2, 3], and with the increasing elderly population, there is concern that the incidence of these fractures could increase [4].

For managing humeral shaft fractures, a functional brace is the preferred method because it offers advantages such as early resumption of activities, favourable functional outcomes, limited complications, patient comfort, and cost-effectiveness [5, 6]. Initially, a coaptation splint is used for 7 to 14 days to reduce swelling, followed by the application of a humeral functional brace. Regular radiographs are taken over three weeks to ensure proper maintenance of reduction, with subsequent imaging obtained at 3- to 4-week intervals [7–9].

Recent surgical techniques and implant innovations have led to increased inclination toward immediate intervention for humeral shaft fractures [4, 10]. Despite the presence of published randomized controlled trials, the question of whether surgical treatment yields superior or inferior outcomes compared to nonoperative management for humeral shaft fractures remains unresolved [11, 12]. High-income countries show comparable functional outcomes and patient satisfaction between surgical and nonsurgical management of humeral midshaft fractures, while low-income countries prefer nonoperative management of humeral shaft fractures, but concerns about elbow stiffness persist [13–15].

Most of the available studies on functional outcomes after nonoperative management of humeral shaft fractures have involved the use of a functional brace, which is not commonly used in low-income settings. This study aimed to assess the efficacy of nonoperative treatment for humeral shaft fractures using a U-shaped slab, a common approach in low-income settings.

## Methodology

### Study design and settings

This prospective cohort study focused on participants with humeral shaft fractures who sought consultation between August 2021 and August 2022. The study was conducted within the orthopedic unit of the Department of Surgery at the University Teaching Hospital of Kigali (CHUK) and Rwanda Military Hospital (RMH). These hospitals, CHUK and RMH, are tertiary and referral public hospitals in Rwanda that cater to patients from across the country. Both are situated in Kigali, the capital city of Rwanda.

CHUK has a total of 519 beds for inpatients, with the surgery department occupying 125 beds, 40 (32%) of which are designated for orthopedics. On the other hand, the RMH has a bed capacity of 500, providing healthcare services to approximately 40,000 to 50,000 patients annually, including both military personnel and civilians.

### Study population and eligibility criteria

This study included individuals aged 16 years or older who had sustained humeral shaft fractures suitable for nonoperative management (less than 20 degrees of anteroposterior angulation, less than 30 degrees of varus-valgus angulation and less than 3 cm of shortening where only limb traction done during splint application. Participants sought consultation at the CHUK or RMH within two weeks of injury during the study period and underwent conservative treatment involving a U-shaped slab. Patients with unacceptable alignment for nonoperative treatment; those with nonunion or malunion; those with a floating elbow, pathological fractures, or a history of osteomyelitis; and those with open fractures or burns that impeded nonoperative treatment were excluded.

### Study procedure

Patients with acute closed humeral shaft fractures who sought consultation at the outpatient department (OPD) or Accident and Emergency Departments and were prescribed nonoperative treatment were included in the study. Patients were enrolled after receiving initial treatment (only limb traction during splint application and weight of the splint itself creates a continuous gentle pulling force on the limb which helped to maintain fracture reduction) there clinically and radiologically follow-up at six and twelve weeks in addition patients were encouraged for self-exercise as pain tolerated by moving limb and joints as much as possible.

At the 6-week follow-up, x-ray controls were included. For patients who exhibited both clinical and radiological signs of union, the U-shaped slab was removed, and physiotherapy was prescribed two times per week for a period of six weeks. The assessment at the 6-week follow-up included evaluating radiological and clinical signs of union indicated by absence of bone pain, tenderness when stressing the fracture site, as well as joint movement. At the 12-week follow-up, another x-ray was taken, and the functional outcome was assessed and patients noted to have non-union offered surgery.

Treatment outcomes were evaluated using specific parameters, including alignment, consolidation, complications, International Classification of Impairments, Disabilities, Handicaps, and DASH score. Alignment measurements were conducted in both the coronal plane (varus and valgus) and the sagittal plane (anterior and posterior) using Dx-view and Vision web computerized systems. These measurements were derived from both initial and final radiographs. Consolidation was clinically evaluated and characterized by the absence of bone pain, tenderness, and movement when stressing the fracture site. Radiographic union was determined by the presence of callus formation on plain x-rays. Delayed union was defined as the absence of clinical union 12 weeks after the initial trauma.

Limb function was assessed by evaluating pain and the return of movement at the shoulder, elbow, and hand. This assessment was graded according to the International Classification of Impairments, Disabilities, and Handicaps as follows:

- Grade I: Pain and complete limitations preventing any activities.
- Grade II: Mild pain and significant restraint, severely impeding daily activities.
- Grade III: Limitations allowing for daily activities with some challenges.
- Grade IV: Minimal restriction, no interference with daily activities, and absence of pain.
- Grade V: Unrestricted activities and absence of pain

### Data collection and analysis

In this study, patients with humeral shaft fractures from the outpatient department (OPD) or from the Accident & Emergency Department were identified, and relevant information was recorded on data capture forms. A preelaboration questionnaire was completed at the 6th and 12th weeks of follow-up. The data were entered into EpiData and secured on the primary investigator’s password-protected computer.

When collecting the data, we categorized the energy mechanism as follows:

- Low-Energy Mechanism: Humeral shaft fractures result from relatively mild or minimal forces applied to the humerus. These fractures typically occur during activities or incidents with minimal impact on the arm, such as slipping, tripping, or falling from a standing position without significant external force applied to the arm.
- Moderate Energy Mechanism: Humeral shaft fractures are the result of forces stronger than those causing low-energy fractures but less severe than those associated with high-energy fractures. This category included incidents where patients experienced a direct blow to the arm without substantial impact (this category includes incidents where patients experienced a direct blow to the arm including sport injuries such as football, basketball without substantial impact).
- High Energy Mechanism: Humeral shaft fractures occur due to extremely strong forces or significant trauma and are often linked to severe accidents, falls from considerable heights, or direct blows with substantial impact.

For analysis, the data were analysed with the statistical software package SPSS version 28.0. The study findings are presented in tables and charts. To determine associations within the results, the chi-square test, binary logistic regression test, and multivariable logistic regression test were employed. The significance of the results was assessed by calculating the p value and odds ratio (OR).

## Results

A total of 73 adult patients with humeral shaft fractures who sought consultation during the study period were enrolled, and no patients were lost to follow-up. These individuals were treated using a U-shaped slab, and their progress was monitored for three months. Subsequently, the functional outcomes were assessed.

### Sociodemographic profile of the enrolled participants

The study included participants ranging from 16 to 76 years of age, with an average age of 35 years. Predominantly, the participants were male (73.97%), with a significant portion residing in Kigali city (43.8%). The male‒female ratio was approximately 3 to 1.

Regarding the etiology of humeral shaft fractures, the largest percentage were motorcycle accidents (52.5%), followed by falls (26.03%), motor vehicle accidents (12.33%), assault (6.85%), and bicycle accidents (2.74%). Among the participants, 19.18% had hypertension, while 6.85% had diabetes mellitus. Additionally, 13.7% of the participants had a history of smoking.

**Table:** Sociodemographic profile of enrolled participants

Characteristics	*n*	%
*Age (in years)*		
Median (Q1-Q3)	33 (25–46)	
*Sex*		
Male	54	73.97
Female	19	26.03
*Residence*		
Kigali city	32	43.83
*Eastern*		34.25
Northern	7	9.59
Southern	7	9.59
Westhern	2	2.74
Level of Education	25	
None	7	9.59
Primary	32	43.84
Secondary	32	43.84
University	2	2.74
*Cause of injury*		
Motor cycle accident	38	52.05
Driver	8	21.05
Passenger	24	63.16
Pedestrian	6	15.79
Motor vehicle accident	9	12.33
Rider	1	11.11
Passenger	4	44.44
Pedestrian	4	44.44
Bicycle accident	2	2.74
Passenger	2	66.67
Pedestrian	1	33.33
Fall	19	26.03
Assaults	5	6.85
*Comorbidity*		
Diabetes mellitus	5	6.85
Hypertension	14	19.18
Smoking	10	13.7
Medication history	2	2.74

The median duration between the accident and consultation was two days. A majority of the patients (73.97%) presented with right-sided injuries, and 69.86% of them had injuries to their dominant limb. Regarding the location of the fractures, 75.34% were mid-shaft fractures, while 19.18% and 5.48% were proximal third shaft fractures and distal third shaft fractures, respectively. According to the fracture patterns, the most common type was oblique fracture (53.42%), followed by spiral fracture (28.77%), transverse fracture, and comminuted fracture (9.59% and 8.22%, respectively).

Concerning the energy mechanisms causing the fractures, 49.32% were the result of high-energy forces, while 45.20% and 5.48% were caused by medium-energy and low-energy mechanisms, respectively.

**Table:** Clinical characteristics of the humeral shaft fractures managed non-operatively

Variables	*n*	%
*Time between accident and consultation*		
Median (Q1-Q3)	2 (0–4) days	
*Site of injury*		
Right	54	73.97
Left	19	26.03
*Dominant limb affected*		
Yes	51	69.86
No	22	30.14
*Fracture location*		
Mid-shaft	55	75.34
Proximal third shaft	14	19.18
Distal third shaft	4	5.48
*Pattern of fracture*		
Oblique	39	53.42
Spiral	21	28.77
Transverse	7	9.59
Comminuted	6	8.22
*Mechanism of injury*		
Low energy	4	5.48
Moderate energy	33	45.21
High energy	36	49.32

### Baseline clinical factors

A large proportion of patients had no significant physical examination findings in various areas: shoulder (89.04%), elbow (93.15%), neurovascular (95.89%), or skin (80.82%). Approximately 45.21% of participants demonstrated moderate soft tissue swelling, while 54.79% displayed mild soft tissue swelling. Notably, three patients (4.11%) experienced radial nerve palsy, and the occurrence of abrasions and lacerations in each group was 9.59%.

**Table:** Physical examination findings at presentation

Findings	*n*	%
*Shoulder exam*		
Normal	65	89.04
Swelling	7	9.59
Pain and swelling	1	1.37
*Elbow exam*		
Normal	68	93.15
Swelling	5	6.85
*Neurovascular exam*		
Normal	70	95.89
Radial nerve injury	3	4.11
*Skin status*		
Normal	59	80.82
Abrasions	7	9.59
Lacerations	7	9.59
*Soft tissue status*		
Mild swelling	40	54.79
Moderate swelling	33	45.21

### Clinical factors at the 6th and 12th weeks of follow-up

By assessing the joint range of motion and related discomfort at the 6th week of follow-up, following Stewart and Hundley’s classification, we observed that eight patients (10.96%) achieved an excellent rating, 52 (71.23%) received a good rating, six (8.22%) were evaluated as fair, and seven (9.59%) were categorized as poor. At the 12th week, utilizing the International Classification of Impairments, Disabilities, and Handicaps, a significant majority of the participants achieved favourable functional grades (IV & V) for the affected limb, accounting for 77%. Consequently, the remaining 23% exhibited less favourable functional grades (II and III).

**Figure:**
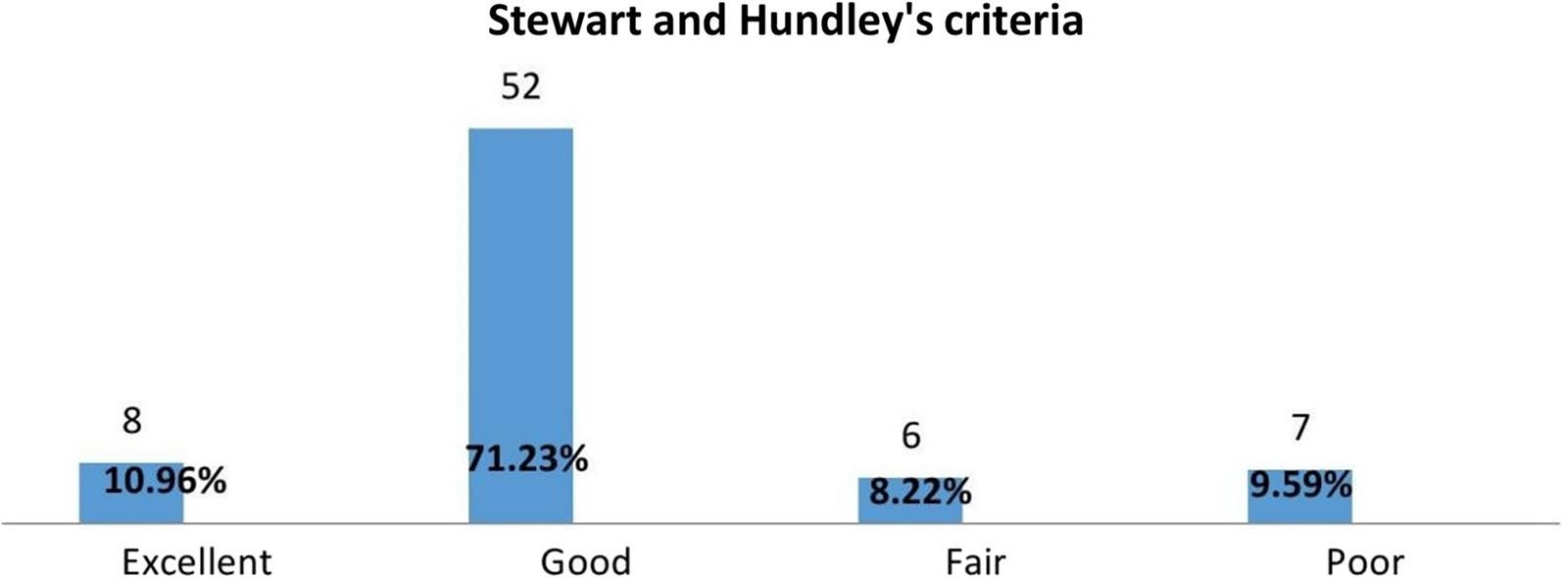
Function grading at the 6thweek of follow-up by Stewart and Hundley's criteria

**Figure:**
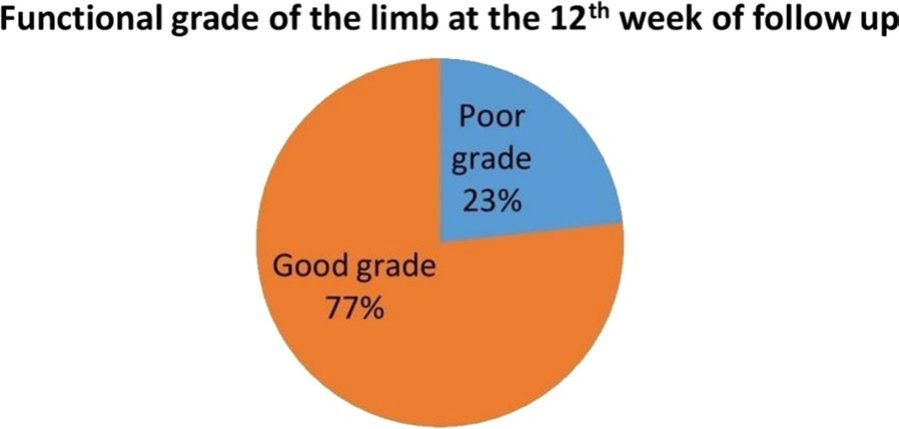
Functional grade of the affected limb at the 12th week of follow-up

### Radiological and clinical findings at 6 and 12 weeks of follow-up

At the 6th week of follow-up, 62 (84.93%) of the participants exhibited adequate callus formation coupled with a lack of pain and tenderness at the site of the fracture. Only 6.85% reported severe pain, while 9.59% experienced moderate pain. The most prevalent complication was elbow pain, affecting 97.26% of the participants, followed by elbow stiffness defined as restriction of range of motion experienced at the elbow joint, typical measured in degree of flexion and extension, noted in 86.30% of the patients. No comparison of affected to unaffected limb done and documented during the study.

During the 12th week of follow-up, the status of the callus improved in 89.04% of the participants, while 10.96% still experienced signs of delayed union of the fracture.

A total of seventy-one participants, accounting for 97.26% of the sample, successfully restarted their activities of daily living (ADL) within twelve weeks. The average duration for initiating ADLs was found to be eight weeks. Notably, none of the participants reported suffering severe pain at this juncture. The majority of participants (53.42%) indicated that they had experienced mild pain. Remarkably, complete functional restoration was observed in all three individuals diagnosed with radial nerve palsy. The calculated median DASH score was 14.12.

**Table:** Radiological and clinical findings at the 6th week of follow-up

Variables	*n*	%
*Presence of adequate callus (bridging both bony cortices)*		
Yes	62	84.93
No	11	15.07
*Absence of pain and tenderness at the fracture site*		
Yes	62	84.93
No	11	15.07
*Pain (Visual Analogue scale)*		
None (0)	2	2.74
Mild (1–3)	59	80.82
Moderate (4–6)	7	9.59
Severe (7–10)	5	6.85
*Complications*		
Elbow pain	71	97.26
Elbow stiffness	63	86.30
Shoulder pain	40	54.79
Shoulder stiffness	19	26.03
Skin injury	4	5.48
Radial nerve injury	3	4.11

**Table:** Radiological and clinical findings at the 12th week of follow-up

Variables	n	%
*The Status of the callus improved*		
Yes	65	89.04
No	8	10.96
*Delayed union of fracture*		
Yes	8	10.96
No	65	89.04
*Joint stiffness*		
Yes	16	21.92
No	57	78.08
*Pain score (Visual Analogue Scale)*		
None (0)	26	35.62
Mild (1–3)	39	53.42
Moderate (4–6)	8	10.96
Severe (7–10)	0	0.00
*Neurovascular exam findings*		
Radial nerve injury	0	0.00
*Started activities of daily living*		
Yes	71	97.26
No	2	2.74
Timing of starting activities of daily living in weeks		
Mean ± SD	8 ± 1.6	
*Group*		
Less than 6 weeks	2	2.82
Between 6 and 8 weeks	31	43.66
Between 9 and 12 weeks	38	53.52
*DASH Score*		
Median (Q1-Q3)	14.12 (3–12)	

**Figure:**
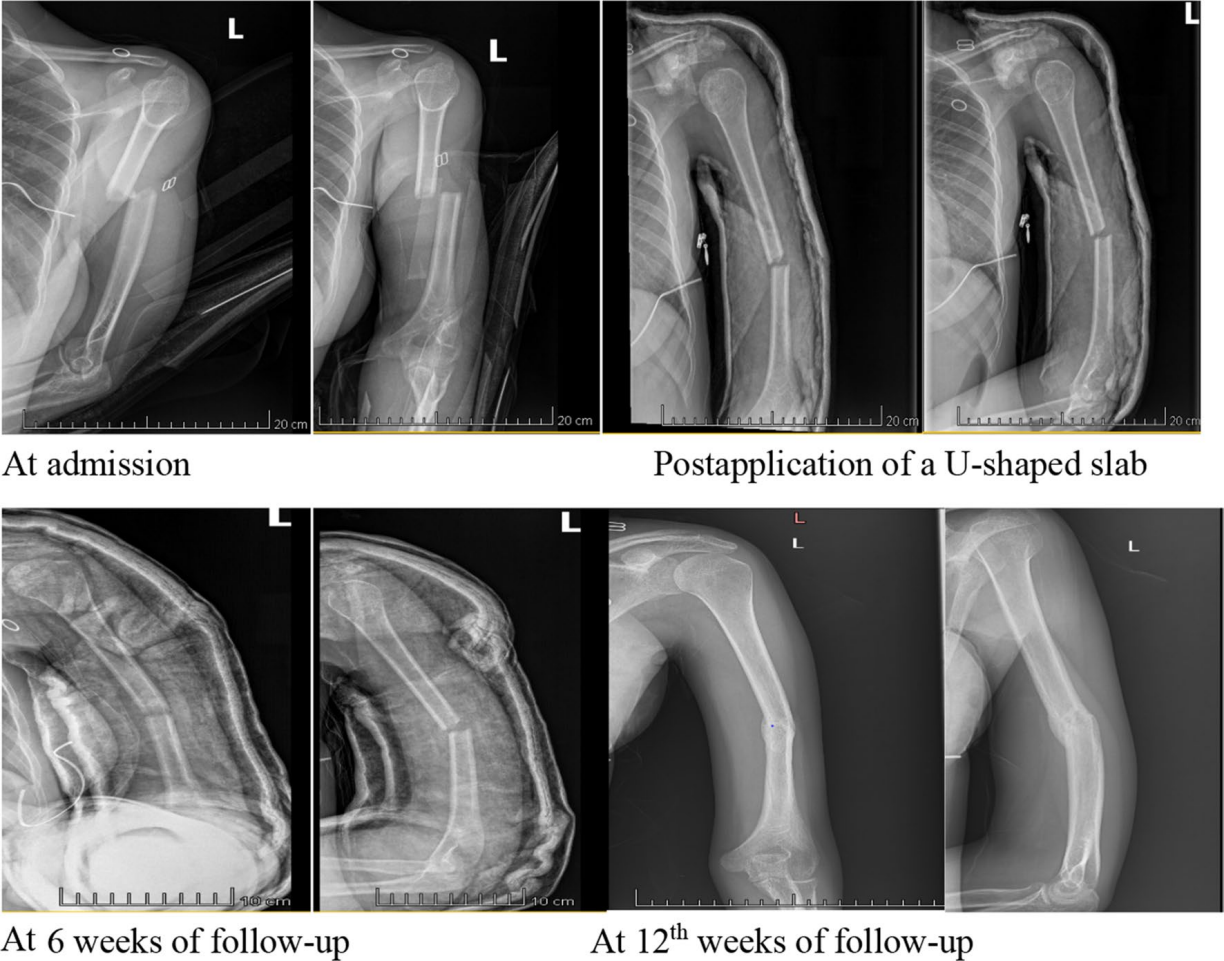
Association between alignment at the 12th week of follow-up and functional outcome

A significant association was found between reduced antero-posterior (AP) angulation at the 12th week of follow-up and favourable functional outcomes. However, there were no statistically significant differences in the outcomes concerning varus/valgus alignment or shortening of the affected limb *p* 0.179.

**Table:** Association between alignment at the 12th week of follow-up and the functional outcome

Alignment at the 12th week	Functional outcome		P value
	Poor grade	Good grade	
*AP angulation (in degrees)*			
Median (Q1-Q3)	8 (5–10)	5 (4–8)	0.022
Mean ± SD	7.64 ± 2.64	6.05 ± 2.92	
*Varus/Valgus (in degrees)*			
Median	5 (4–6)	4.5 (3–5)	0.179
Mean ± SD	5.53 ± 3.35	4.03 ± 1.82	
*Shortening/Overlapping (in cm)*			
Median	0 (0–0)	0 (0–0)	0.336
Mean ± SD	0.06 ± 0.43	0.06 ± 0.16	

### Factors associated with delayed fracture union

When examining the occurrence of delayed fracture union, 19.05% of patients with spiral fractures and 57.14% of patients with transverse fractures experienced this condition (*p* < 0.001).

In terms of medical history, 60% of patients with diabetes mellitus and 42.86% of those with chronic hypertension exhibited delayed fracture union, while considerably smaller percentages of 7.35% and 3.39%, respectively, were affected among patients without these conditions (*p* < 0.001). A similar pattern emerged concerning smoking history, with 60% of patients with such a history experiencing delayed fracture union, in contrast to only 3.17% of those without such a history.

**Table:** Factors associated with delayed fracture union among study participants

Predictors	Delayed fracture union		*P* value
	Yes	No	
*Pattern of fracture*			
Spiral	4 (19.05%)	17 (80.95%)	< 0.001
Oblique	0 (0.00%)	39 (100%)	
Transverse	4 (57.14%)	3 (42.86%)	
Comminuted	0 (0.00%)	6 (100%)	
*Location of fracture*			
Mid-shaft	5 (9.09%)	50 (90.91%)	0.559
Proximal third shaft	2 (14.29%)	12 (85.71%)	
Distal third shaft	1 (25.00%)	3 (75.00%)	
*Age in years*			
< 35	2 (5.41%)	35 (94.59%)	< 0.001
35–65	3 (9.38%)	29 (90.63%)	
> 65	3 (75.00%)	1 (25.00%)	
*Diabetes mellitus*			
Yes	3 (60.00%)	2 (40.00%)	< 0.001
No	5 (7.35%)	63 (92.65%)	
*Hypertension*			
Yes	6 (42.86%)	8 (57.14%)	< 0.001
No	2 (3.39%)	57 (96.61%)	
*Smoking*			
Yes	6 (60.00%)	4 (40.00%	< 0.001
No	2 (3.17%)	61 (96.83%)	

### True predictors of functional outcome using multivariable logistic regression analysis

All the predictors that showed statistically significant associations with functional outcomes in the binary logistic regression analysis, including patient age, pattern of fracture, fracture location, smoking status, mechanism of injury, hypertension, diabetes status, and education level, were incorporated into the multivariable logistic regression analysis. After the final analysis was conducted, patient age, fracture pattern, and smoking status were identified as the most accurate independent predictors of functional outcomes among patients with humeral shaft fractures treated nonoperatively with a U-shaped slab.

Participants younger than 35 years were more likely to attain good functional outcomes than were those aged 35 to 65 years (OR = 5.15; 95% CI = 1.27–20.83; *p* = 0.021). Conversely, all four patients aged older than 65 years exhibited poor functional outcomes by the 12th week of follow-up.

Regarding the fracture patterns, participants with oblique fractures exhibited a significantly greater probability of achieving good functional outcomes by the 12th week of follow-up than did those with transverse fractures (OR = 21.8; 95% CI: 3.18–152.0; *p* = 0.002). Similarly, participants with spiral fractures tended to have better functional outcomes than did those with transverse fractures, although the difference was not significant (OR = 6.25; 95% CI = 0.94–41.52; *p* = 0.058).

**Table:** Patients with no history of smoking were significantly better able to achieve good functional outcomes than were those with a smoking history (OR = 12.36; 95%CI = 2.73–56.08; *p* = 0.001)

Predictors	AOR	95% CI	SE	*P* value
*Age category*				
< 35 years	26.05	1.82–372.99	35.37	0.016
35–65 years	Ref			
> 65 years	–			
*Pattern of fracture*				
Transverse	Ref			
Spiral	15.49	0.68–353.16	24.71	0.086
Oblique	42.02	1.62–1085.1	69.70	0.024
Comminuted	0.79	0.02–23.08	1.36	0.889
*Smoking*				
Yes	Ref			
No	18.08	1.11–293.2	25.71	0.042

AOR: Adjusted Odds Ratio; CI: Confidence Interval; SE: Standard Error, Ref: reference group.

## Discussion

The functional outcomes of humeral shaft fractures treated with a U-shaped slab can vary depending on various factors, including specific fracture characteristics, patient age, overall health, and compliance with rehabilitation and follow-up protocols. Management of humeral shaft fracture with a coaptory U-shaped slab can provide good to excellent outcomes, as revealed by different researchers [16, 17].

In our study, we examined seventy-three participants with humeral shaft fractures (HSFs) whose ages ranged from 16 to 76 years (median age: 33 years). Males constituted the majority of the injured individuals. Among the participants, thirty-seven were younger than 35 years of age, while 32 fell within the 35–65 years age range. Notably, more than half of the participants had sustained their fractures due to motorcycle accidents, followed by falls from heights.

The age group younger than 65 years represents an active demographic group that is predominantly composed of males engaged in various physically demanding occupations. Additionally, given the prevalent use of motorcycles as a primary mode of transportation in our setting, it is unsurprising that motorcycle accidents contribute significantly to the occurrence of injuries in this group. This could explain the higher incidence of injuries among males and the dominant presence of individuals under 65 years old.

Our findings align with a study by Oboirien M. that focused on the management of humeral fractures in a resource-poor region in northwestern Nigeria. Oboirien’s study similarly highlighted the prominence of road traffic accidents, particularly motorcycle accidents, as the leading cause of humeral fractures. This parallel strengthens the consistency and relevance of our observations [18].

Our study revealed interesting patterns concerning the energy mechanisms responsible for humeral shaft fractures (HSFs) across different age groups. We found that more than half of the patients younger than 35 years sustained their fractures through high-energy mechanisms. In the 35- to 65-year-old age group, the majority of patients required moderate to high energy to fracture their humerus. Conversely, most patients aged older than 65 years Experience humeral fractures due to low-energy mechanisms.

Our findings are consistent with the findings of previous studies by G. Tytherleigh-Strong et al., Eben A. Carroll et al., and Nicolas Gallusser. These studies also revealed a bimodal age distribution pattern for HSF. The first peak occurred in the third decade among men and was characterized by high-energy mechanisms, while the second peak was observed in women during the sixth to seventh decade and was typically caused by low-energy mechanisms [8, 19, 20].

The rate of union in our study was 89.04%, while 10.96% experienced delayed union. Our study’s union rate aligned with similar findings by Ghadeer H. Majeed et al. (90.9%) and L. Klenerman et al. (90.8%) [16, 21] but was lower than the findings of Abdul Rehman et al. (98%) and Hunter (93.4%) [22, 23].

Notably, we observed that most participants with transverse fracture patterns developed delayed union, with half of all participants with transverse fractures experiencing this outcome. This observation echoes the results of L. Klenerman, who reported a strong association between delayed union and transverse mid-shaft humeral shaft fractures, with three out of five delayed union cases involving transverse fractures [21].

In our study, eleven patients showed no signs of union during the 6th week of follow-up. Eight (72.73%) patients continued to exhibit a lack of clinical and radiological improvement in union signs during the 12th week of follow-up. At 6th week of follow-up those who exhibited lack of imorovement the splint was readjusted resulting into improved angulations as the splint’s weight continued effectively in reduction of the fracture. These findings are consistent with the results of Sargeant et al., who suggested that the absence of clinical and radiological signs of union at the 6th week of follow-up can predict delayed union [24].

The angular deformities of humeral shaft fractures gradually improved throughout treatment with the U-shaped slab. Upon admission, the median anterior/posterior angulation was 10.50°, while the median varus/valgus angulation was 8.17°. By the 12th week of follow-up, these angles improved, with the median anterior/ posterior angulation decreasing to 6.2 degree and median varus/valgus angulation decreased to 4.38 degree. Although most participants exhibited some remaining angular deformities even after healing, these deformities did not have a substantial impact on the final functional outcome. Our findings are consistent with similar research by H. Majeedet et al., L. Klenerman, and Abdul Rehman et al., who also demonstrated that despite residual angular deformities, overall functional outcomes were not adversely affected [16, 21, 22].

The incidence of radial nerve palsy in our study was 4.11%. Notably, patients with primary radial nerve palsy spontaneously recovered recovered by the 12th week of follow-up, and no patients developed radial nerve palsy. After receiving treatment. Our study’s results were more favourable than those of a systematic review conducted by Y. C. Shao et al. on radial nerve palsy associated with humeral shaft fractures. In that review, the prevalence of radial nerve palsy across 21 papers was 11.8% (532 palsy cases among 4517 fractures). Additionally, most palsies (70.7%) recovered spontaneously in patients treated conservatively. The lower incidence of radial nerve palsy in our study can be attributed to the smaller sample size, as the systematic review included a significantly larger number of participants [25].

In line with our findings, Hunter also reported that 8.5% of patients with radial nerve palsy achieved spontaneous recovery by the 12th week. The positive recovery trend observed in both studies highlights the potential for favourable outcomes in patients with radial nerve palsy associated with humeral shaft fractures [23].

In a study conducted by Abdul Rehman et al. involving 100 patients with humeral shaft fractures (HSFs), similar criteria were employed. Their results indicated that 60% of patients achieved an excellent outcome, 27% obtained a good outcome, 11% had a fair outcome, and 2% experienced a poor outcome. Notably, Abdul Rehman’s study evaluated outcomes at the 16th week of follow-up, in contrast to our investigation, which assessed outcomes at the 6th week immediately after U-slab removal. Interestingly, a parallel comparison between the two studies revealed a consistent trend. Both studies reported that a substantial proportion of patients achieved excellent to good outcomes (87% in Abdul Rehman’s study and 81.19% in our study).

We did not find a discernible association between sex and functional outcomes. However, several factors, including patient age, fracture patterns, fracture location, smoking habits, and adherence to rehabilitation protocols, emerged as statistically significant predictors of patient outcomes.

Among our participants, 10 were smokers, and 63 were nonsmokers. Within the smoker group, a majority experienced delayed union and exhibited poor functional grades. Moreover, all patients who did not adhere to the rehabilitation instructions demonstrated poor functional outcomes. In contrast, only 5% of those who followed the rehabilitation instructions experienced poor functional outcomes. Our rehabilitation protocol consists of self-exercise as pain tolerated by moving limb and joints as much as possible and physiotherapy of the affected limb two times per weeks for the period of six weeks.

Our findings are consistent with the findings of E. Shields et al. (2015), who demonstrated that patient age, psychiatric history, insurance type, fracture location, and Charlson comorbidity index score had substantial influences on patient-reported functional outcomes after treating humeral shaft fractures [26].

## Conclusion

We analysed 73 adult patients with closed humeral shaft fractures (HSFs) managed nonoperatively using a U-shaped slab. The median age of the participants was 33 years, with males comprising the majority of the participants and a male-to-female ratio of 3:1. The right side was more commonly affected, and the dominant limb was frequently injured. The majority of patients had mid-shaft fractures, most of which were oblique fractures. Notably, for patients aged older than 65 years, HSF was more commonly associated with low-energy mechanisms, while in younger age groups, moderate-to-high-energy mechanisms were the predominant cause.

We achieved an excellent rate of union with favourable functional outcomes. Delayed union was primarily linked to transverse fracture patterns and a history of smoking. The rate of radial nerve palsy was low, with all patients classified as having primary radial nerve palsies exhibiting neuropraxia. These patients spontaneously recovered within three months, and no patient developed radial nerve palsy after receiving treatment.

Despite some participants healing with the remaining angular deformities, these deformities did not significantly impact the overall functional outcome. The median DASH score was 14.12, reflecting favourable limb function, and the scores ranged from excellent to good. In our analysis, we did not find any association between sex and functional outcome. However, we identified several predictors of functional outcome, including patient age, fracture pattern, history of smoking, and the use of physiotherapy.

Proper patient selection by an orthopaedic surgeon for certain group patients (transverse fracture, with smoking history and older age) and accurate education concerning rehabilitation may offer additional value in improving functional outcomes for patients with humeral shaft fractures managed nonoperatively using a U-shaped slab.

## Limitations of the study

This study has limitations, such as a short follow-up period and small sample size, which may affect the accuracy of its findings. However, a longer follow-up and larger sample size could provide more comprehensive insights into functional outcomes in patients with humeral shaft fractures, enhancing their validity.

## Appendix 1

### Data collection form

At the initial visit

I) Patient details

II) Injury details

III) Known comorbidities/risk factors

IV) Radiological evaluation

V) Physical examination

The 6th week of follow-up

I. Radiological signs of union

II. Clinical signs of union

III. Pain-score

IV. Joints status

**Table:** Stewart and Hundley criteria

Result	Pain	Limitation of elbow or shoulder mobility	Angulations
Very good	No pain	Complete freedom of movement in both the elbow and shoulder	Good radiological alignment
Good	Occasional pain	A restriction of less than 20 degrees in the movement of either the elbow or shoulder	Less than 10^0^
Fair	Activity related pain	A limitation of movement ranging from 20 to 40 degrees in either the elbow or shoulder	Greater than 10^0^
Poor	Constant pain	A limitation of more than 40 degrees in the movement of either the elbow or shoulder	X-ray confirmed nonunion

V. Complications

At the 12th week of follow-up

**Table:** I. Fill dash score

	No difficulty	Mild difficulty	Moderate difficulty	Severe difficulty	Unable
Loosen a tightly closed or a newly sealed flask or bottle	**1**	**2**	**3**	**4**	**5**
Compose text	**1**	**2**	**3**	**4**	**5**
Rotate a key	**1**	**2**	**3**	**4**	**5**
Cook a meal	**1**	**2**	**3**	**4**	**5**
Operate a heavy door by either opening or pushing it	**1**	**2**	**3**	**4**	**5**
Position an item on a shelf that is positioned above your head	**1**	**2**	**3**	**4**	**5**
Engaging in strenuous household tasks, such as cleaning walls or mopping floors	**1**	**2**	**3**	**4**	**5**
Engage in gardening or outdoor yard activities	**1**	**2**	**3**	**4**	**5**
Arranging a bed	**1**	**2**	**3**	**4**	**5**
Transporting a shopping bag or a briefcase	**1**	**2**	**3**	**4**	**5**
Lifting and transporting a substantial item (weighing more than 5 kg)	**1**	**2**	**3**	**4**	**5**
Replacing an overhead light bulb	**1**	**2**	**3**	**4**	**5**
Cleanse or use a hairdryer on your hair	**1**	**2**	**3**	**4**	**5**
Clean the back	**1**	**2**	**3**	**4**	**5**
Wear a pullover sweater	**1**	**2**	**3**	**4**	**5**
Utilizing a knife for food cutting	**1**	**2**	**3**	**4**	**5**
Leisurely activities that demand minimal exertion like playing cards	**1**	**2**	**3**	**4**	**5**
Recreational activities in which you take some force or impact through your arm, shoulder, or hand (eg., hammering, tennis, golf)	**1**	**2**	**3**	**4**	**5**
Recreational activities in which you move your arm freely (eg. throw ball etc.)	**1**	**2**	**3**	**4**	**5**
Manage transportation needs	**1**	**2**	**3**	**4**	**5**
Sexual activities	**1**	**2**	**3**	**4**	**5**
Over the past week, how much has your arm, shoulder, or hand issue impacted your ability to engage in your usual social interactions with family, friends, neighbors, or groups?	**1**	**2**	**3**	**4**	**5**
In the past week, did your arm, shoulder, or hand problem restrict your ability to work or engage in your daily activities?	**1**	**2**	**3**	**4**	**5**
Arm, shoulder or Hand pain	**1**	**2**	**3**	**4**	**5**
Do you experience pain in your arm, shoulder, or hand when engaging in any particular activity?	**1**	**2**	**3**	**4**	**5**
Do you have tingling sensations in your arm, shoulder, or hand?	**1**	**2**	**3**	**4**	**5**
Are you currently encountering a lack of strength in your arm, shoulder, or hand?	**1**	**2**	**3**	**4**	**5**
Do you have stiffness in your arm, shoulder, or hand?	**1**	**2**	**3**	**4**	**5**
Over the last week, to what extent has your ability to sleep been affected due to pain in your arm, shoulder, and hand?	**1**	**2**	**3**	**4**	**5**
I experience reduced capability, confidence, or usefulness due to my arm, shoulder, and hand	**1**	**2**	**3**	**4**	**5**

II. Radiological assessment of fracture

V. Physiotherapy done

VI. Activity of daily living

When have you started activities of daily living (such as washing, cooking, housecleaning, and cleaning yourself)?

VII: Neurovascular Status

VIII. Limb function grading
